# Provisions Relating to Hospitals and Institutions

**Published:** 1921-09-03

**Authors:** 


### Dangerous Drugs Act, 1920.

### PROVISIONS RELATING TO HOSPITALS AND INSTITUTIONS.

This Act came into force on September 1, 1921. The Home Secretary has issued a general permit, exempting hospitals, infirmaries, asylums, Poor-Law institutions, etc., from the Regulations, provided that the drugs are dispensed by a doctor, pharmacist, or dispenser approved by the Ministry of Health, and that the conditions of Schedule 1 of the Order are complied with. If there is no doctor or dispenser exemption is granted subject to Schedule 2 conditions. Exemptions may be revoked, either generally or in an individual case. An important provision is that any hospital or institution exempted must be open to inspection by persons appointed by the Home Secretary. The following is a summary of the requirements under the two schedules mentioned, so far as they affect hospitals and institutions:

### Schedule 1.

1. Supplies only obtainable on order signed by medical officer or pharmacist. Telephone orders not legal, nor is an order signed only by house governor, steward, etc.
2. Persons dispensing, to keep drugs and be responsible. All supplies to be entered in drug ledger, each drug separately.
3. Prescriptions on bed-cards to be in writing, dated, and signed or initialled by doctor, and to give patient's name or reference. When further supply required a new prescription must be given.
4. Persons dispensing prescriptions shall stamp or mark prescription as evidence that it has been dispensed, and keep record of date, names of prescriber and patient.
5. All prescriptions to be kept for two years.
6. Preparations for ward stock only obtainable on written requisition of sister-in-charge, who must keep supply locked up and use by direction of doctor only; these requisitions to be marked "supplied" and filed in dispensary, the ward sister keeping a duplicate. Steps to be taken to prevent theft during transit to the wards.

### Schedule 2.

These Regulations apply to small hospitals with no resident medical officer or dispenser. Under them supplies are obtainable on the order of a doctor attending hospital. Matron receives and keeps supplies locked up and enters particulars in a book—drugs for use on doctor's order only.

### The Drugs Affected.

The drugs affected by the Act are morphine, cocaine, ecgonine, heroin, and salts of these, and opium or preparations containing one-fifth per cent. or more of morphine or one-tenth per cent. of cocaine, ecgonine, or heroin. All medicines diluted to contain lesser percentages of the above are exempted totally. Seventeen preparations which cannot be used for "doping" are also exempted, although they would, by reason of their strength, come within the Act.

A record of the drugs already in stock is not required. Only supplies received on or after September 1, 1921, are dealt with.

No record of preparations made in the dispensary, such as morphia solution, etc., appears to be required from hospitals, and unless sent out in strengths covered by the Act the system in vogue at present may remain unaltered.

The scheme should work fairly easily in hospital practice, but we would like to have seen some provision made for ordering urgent supplies by telephone or wire to meet the needs of outlying hospitals.

